# Real-time data for estimating a forward-looking interest rate rule of the ECB

**DOI:** 10.1016/j.dib.2017.10.025

**Published:** 2017-10-17

**Authors:** Tilman Bletzinger, Volker Wieland

**Affiliations:** Chair of Monetary Economics, Institute for Monetary and Financial Stability, Goethe-University Frankfurt, House of Finance, Theodor-W.-Adorno-Platz 3, 60323 Frankfurt, Germany

**Keywords:** Interest rate rule estimation, Real-time data, Forward-looking data

## Abstract

The purpose of the data presented in this article is to use it in ex post estimations of interest rate decisions by the European Central Bank (ECB), as it is done by Bletzinger and Wieland (2017) [1]. The data is of quarterly frequency from 1999 Q1 until 2013 Q2 and consists of the ECB's policy rate, inflation rate, real output growth and potential output growth in the euro area. To account for forward-looking decision making in the interest rate rule, the data consists of expectations about future inflation and output dynamics. While potential output is constructed based on data from the European Commission's annual macro-economic database, inflation and real output growth are taken from two different sources both provided by the ECB: the Survey of Professional Forecasters and projections made by ECB staff. Careful attention was given to the publication date of the collected data to ensure a real-time dataset only consisting of information which was available to the decision makers at the time of the decision.

**Specifications Table**

**Table:** 

Subject area	*Economics*
More specific subject area	*Monetary Policy, Interest Rate Rules*
Type of data	*Figures, Excel-File*
How data was acquired	*Constructed based on data collected in the internet*
Data format	*Raw, constructed*
Data accessibility	The data is made available in the supplementary material coming with this article.

**Value of the data**

• Real-time data of key macroeconomic variables of the euro area which allows an empirical assessment of monetary policy decisions based on data available to policy makers at the time of the decision.
• Data is not distorted by later revisions based on hindsight information.
• Either the raw data or the constructed data are constant-horizon forecasts of macroeconomic variables which allows an assessment of forward-looking monetary policy decisions.
• Two different sources to construct the same set of forecast variables allows to check which data performs better in empirical assessments of policy decisions.

## 1 Data

Bletzinger and Wieland [1] analyse to what extent the interest rate set by the European Central Bank (ECB) can be explained by means of a simple interest rate rule. Similar to the Taylor [3] rule, it is assumed that the ECB sets its policy rate, the interest rate on its main refinancing operations (MRO), in response to aggregate inflation and output developments in the euro area. While inflation is measured as year-on-year percentage changes using Eurostat's Harmonised Index of Consumer Prices (HICP), output developments are based on the output gap which is defined as the difference between the quarter-on-quarter growth rate of the real gross domestic product and the quarter-on-quarter growth rate of real potential output. It is further assumed that the ECB policy makers are forward-looking and hence react to forecasts of inflation and output developments when setting the contemporaneous interest rate. Based on data availability, the forecast horizon for inflation is three quarters and for the output gap two quarters ahead. To proxy the information and expectation set of the ECB policy makers two different sources are used to construct the quarterly forecast data as outlined below.

## 2 Experimental design, materials and methods

One important aspect of the analysis by Bletzinger and Wieland [1] is the ex post evaluation of policy decisions taken in the past. For a sound analysis, which only takes into account that information which was available to policy makers at the time each decision was made, it is essential to employ real-time data. Hence, careful attention was given to the publication date of the collected data. In this regard, the policy rate set by the ECB policy makers in the second month of each quarter is used and all other variables are chosen such that they have been available at the time of the decision. The policy rate is the interest rate on main refinancing operations (MRO) irrespective of the tender procedure implemented by the ECB (variable or fixed rate tenders).

The benchmark model in Bletzinger and Wieland [1] makes use of forecasts for inflation and output growth based on the Survey of Professional Forecasters (SPF) provided by the ECB. This data is readily available with quarterly frequency in a constant-horizon forecast format (three quarters ahead for inflation and two for output growth) such that it can be directly employed in the analysis. Following Orphanides and Wieland [2], the growth rate of potential output is calculated based on the semi-annual forecasts provided by European Commission in its annual macro-economic (AMECO) database. To convert the AMECO forecasts of potential output with annual frequency into constant-horizon growth rate forecasts of two quarters ahead, first yearly growth rates are calculated which are then used to interpolate quarterly numbers with the weights illustrated in Table 1. While such an averaging procedure is not able reproduce the quarterly dynamics underlying the annual numbers, it is noted that potential output is relatively stable and adjusts only sluggishly. Hence, large quarterly dynamics are not to be expected anyway. In addition to the correct weights, as explained above due to real-time considerations, it is important to use the most recently available forecast data at the time of each policy decision. As the AMECO database is only updated semi-annually, the most recent vintage is valid for two consecutive quarters. Contrary to that, the SPF forecasts are already published in real-time without later revisions.

**Table 1:** Weights to convert annual forecasts into constant-horizon forecasts two-quarters ahead.

**Time of policy decision**	**Two-quarters ahead forecast**	**Weight for annual 1998 data**	**Weight for annual 1999 data**	**Weight for annual 2000 data**	**…**
1999:Q1	1999:Q3	0.25	0.75	0.00	…
1999:Q2	1999:Q4	0.00	1.00	0.00	…
1999:Q3	2000:Q1	0.00	0.75	0.25	…
1999:Q4	2000:Q2	0.00	0.50	0.50	…
2000:Q1	2000:Q3	0.00	0.25	0.75	…
…	…	…	…	…	…

The alternative model of Bletzinger and Wieland [1] uses the quarterly ECB staff macroeconomic projections as forecasts for inflation and output. Similar to the potential output data by AMECO, the staff projections of the inflation rate and real output growth are only available for a longer observation period with annual frequency. Hence, to obtain constant-horizon forecasts the same weighted average approach as explained in Table 1 is employed. For inflation is important to note that the weights in Table 1 have to be shifted one row up as the horizon is three quarters ahead. Contrary to potential ouptut, the lack to reproduce quarterly dynamics based on annual numbers is an obvious flaw when applying this methodology to more dynamic variables such as inflation and output growth.

**Fig. 1:**
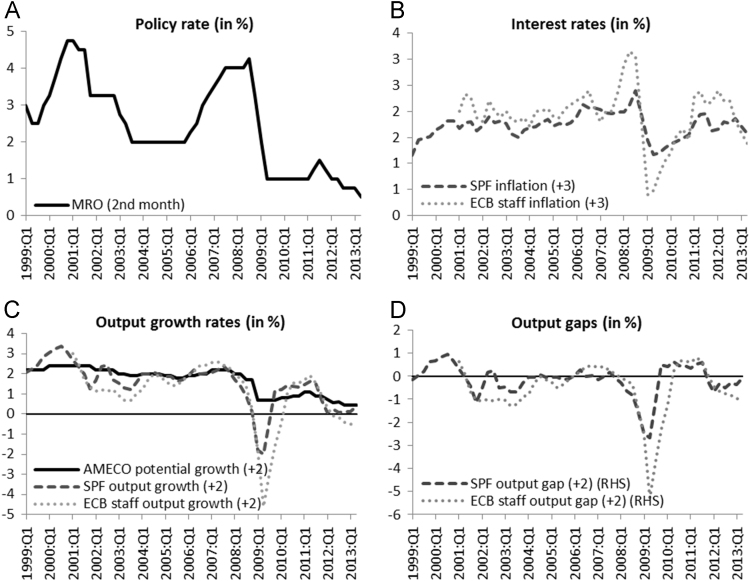
Data used in the estimation of Bletzinger and Wieland [1].

The full dataset containing the final data for the interest rate, inflation rate (SPF and ECB staff), real output growth rate (SPF and ECB staff) and potential output growth rate which are used in the estimation of Bletzinger and Wieland [1] can be found in the attached Excel-file. The sample ranges from 1999 Q1 until 2013 Q2 and is visualised in Fig. 1.

The projection exercises in Bletzinger and Wieland [1] which use the estimated interest rate rule to obtain prescriptions of future policy rates are based on the same structural equation. As the contemporaneous interest rate is explained using forecasts of inflation and the output gap, the future projections of the policy rate rely on forecasts of future forecasts of inflation and the output gap. Based on the law of iterated expectations, the best forecast of a future forecast of a variable is simply today's forecast of that variable. Hence, to obtain constant-horizon forecasts into the future, the same weighted average approach as shown in Table 1 is applied to the three-quarter and seven-quarter-ahead forecasts of inflation and to the two-quarter and six-quarter-ahead forecasts of real output growth available in the SPF. Likewise, the weights are shifted accordingly to obtain future forecasts of AMECO's potential output growth rate.
