# Supplementary material for: Real-time data for estimating a forward-looking interest rate rule of the ECB
Source: Data Brief. 2017 Oct 17;15:687–90. doi: 10.1016/j.dib.2017.10.025 (PMC5671468; doi:10.1016/j.dib.2017.10.025)
Supplement: Supplementary file 1 — Supplementary material [file mmc1.pdf]

Tilman Bletzinger

Volker Wieland

Frankfurt, October 2 , 2017

Regarding both authors we wish to confirm that there has been no significant financial support for this work that could have influenced its outcome.

Regarding Volker Wieland we wish to confirm that there are no known conflicts of interest associated with this publication that could have influenced ist outcome.

Regarding Tilman Bletzinger we wish to draw the attention of the Editor to the following facts. Tilman Bletzinger has been hired as economist by the European Central Bank. The decision making of this institution is the subject of our research study. However, this research was conducted when Tilman Bletzinger was a doctoral researcher at Goethe University. Thus, it was conducted prior to Tilman Bletzinger joining the ECB and prior to when it could have been known that he would receive a job offer from the ECB. Hence, no conflict of interest could have influenced the outcome of our analysis.

Volker Wieland   Tilman Bletzinger
